# Reference Grade Characterization of Polymorphisms in Full-Length HLA Class I and II Genes With Short-Read Sequencing on the ION PGM System and Long-Reads Generated by Single Molecule, Real-Time Sequencing on the PacBio Platform

**DOI:** 10.3389/fimmu.2018.02294

**Published:** 2018-10-04

**Authors:** Shingo Suzuki, Swati Ranade, Ken Osaki, Sayaka Ito, Atsuko Shigenari, Yuko Ohnuki, Akira Oka, Anri Masuya, John Harting, Primo Baybayan, Miwako Kitazume, Junichi Sunaga, Satoko Morishima, Yasuo Morishima, Hidetoshi Inoko, Jerzy K. Kulski, Takashi Shiina

**Affiliations:** ^1^Division of Basic Medical Science and Molecular Medicine, Department of Molecular Life Science, Tokai University School of Medicine, Isehara, Japan; ^2^Molecular Biology Applications, Pacific Biosciences, Inc, Menlo Park, CA, United States; ^3^Pacific Biosciences Division, Tomy Digital Biology Co., Ltd, Tokyo, Japan; ^4^The Institute of Medical Sciences, Tokai University, Isehara, Japan; ^5^GenoDive Pharma Inc., Atsugi, Japan; ^6^Division of Endocrinology, Diabetes, and Metabolism, Hematology, Rheumatology (Second Department of Internal Medicine), Graduate School of Medicine, University of the Ryukyus, Nishihara, Japan; ^7^Department of Promotion for Blood and Marrow Transplantation, Aichi Medical University School of Medicine, Nagakute, Japan; ^8^School of Psychiatry and Clinical Neurosciences, The University of Western Australia, Perth, WA, Australia

**Keywords:** human leukocyte antigen, HLA, next-generation sequencing, NGS, SMRT sequencing, genotyping, PacBio RS II, Ion PGM

## Abstract

Although NGS technologies fuel advances in high-throughput HLA genotyping methods for identification and classification of HLA genes to assist with precision medicine efforts in disease and transplantation, the efficiency of these methods are impeded by the absence of adequately-characterized high-frequency HLA allele reference sequence databases for the highly polymorphic HLA gene system. Here, we report on producing a comprehensive collection of full-length HLA allele sequences for eight classical HLA loci found in the Japanese population. We augmented the second-generation short read data generated by the Ion Torrent technology with long amplicon spanning consensus reads delivered by the third-generation SMRT sequencing method to create reference grade high-quality sequences of HLA class I and II gene alleles resolved at the genomic coding and non-coding level. Forty-six DNAs were obtained from a reference set used previously to establish the HLA allele frequency data in Japanese subjects. The samples included alleles with a collective allele frequency in the Japanese population of more than 99.2%. The HLA loci were independently amplified by long-range PCR using previously designed HLA-locus specific primers and subsequently sequenced using SMRT and Ion PGM sequencers. The mapped long and short-reads were used to produce a reference library of consensus HLA allelic sequences with the help of the reference-aware software tool LAA for SMRT Sequencing. A total of 253 distinct alleles were determined for 46 healthy subjects. Of them, 137 were novel alleles: 101 SNVs and/or indels and 36 extended alleles at a partial or full-length level. Comparing the HLA sequences from the perspective of nucleotide diversity revealed that HLA-DRB1 was the most divergent among the eight HLA genes, and that the HLA-DPB1 gene sequences diverged into two distinct groups, DP2 and DP5, with evidence of independent polymorphisms generated in exon 2. We also identified two specific intronic variations in HLA-DRB1 that might be involved in rheumatoid arthritis. In conclusion, full-length HLA allele sequencing by third-generation and second-generation technologies has provided polymorphic gene reference sequences at a genomic allelic resolution including allelic variations assigned up to the field-4 level for a stronger foundation in precision medicine and HLA-related disease and transplantation studies.

## Introduction

The Major Histocompatibility Complex (MHC) comprising HLA class I and class II molecules (antigens) are polymorphic cell-membrane-bound glycoproteins that regulate the immune response by presenting peptides of fragmented proteins to circulating cytotoxic and helper T lymphocytes, respectively. These peptide-presenting-antigens are encoded by the genomic regions within the MHC that in humans are known specifically as the Human Leukocyte Antigen (HLA) class I and class II gene loci. The HLA molecules are investigated continuously due to their crucial role in the regulation of innate and adaptive immune responses (1, 2), during rejection, graft-versus-host disease (GVHD) of hematopoietic stem cell transplants (3, 4) and the pathogenesis of numerous infectious and/or autoimmune diseases (5–8).

Conventional PCR-based genotyping approaches incorporating restriction fragment length polymorphism (PCR-RFLP) (9), single strand conformation polymorphism (PCR-SSCP) (10), sequence-specific oligonucleotides (PCR-SSO) (11), sequence-specific primers (PCR-SSP) (12), and Sanger sequencing-based typing (PCR-SBT) (13) have been used for HLA-testing in disease association and pre-transplantation (14–16) analysis. However, these methods are limited in their ability to decipher chromosomal phase (*cis/trans*) ambiguity and/or imprecise allele identification (17, 18) and may leave multiple pairs of HLA gene alleles unresolved. Moreover, the traditional methods focus only on the variations in the highly polymorphic regions of the HLA genes, and thus the majority of the assays only interrogate exons 2 and 3 or exons 2, 3, and 4 of the class I loci and exons 2 or exons 2 and 3 of class II. As a result, the genetic variations in the non-coding regions that regulate RNA expression levels (19–21), or the exonic coding sequences outside of the polymorphic domains have remained largely ignored. On the other hand, methods that use full-length gene sequences including the promoter-enhancer region, 5′ and 3′ untranslated regions (UTRs), as well as all exonic and intronic regions for HLA genotyping, would be more accurate for the discovery of new polymorphisms associated with disease susceptibility and transplantation outcome. To this end, however, not more than 10% of ~ 17,700 known alleles in the IPD-IMGT/HLA reference database [Release 3.31, https://www.ebi.ac.uk/ipd/imgt/hla/, (22)] are classified as full-length HLA gene sequences. Consequently, a comprehensive collection and classification of full-length HLA allele sequences including information from the “undetermined” regions of the HLA genes are desired to promote the future development of more accurate and reliable HLA genotyping methods.

**Figure 1:**
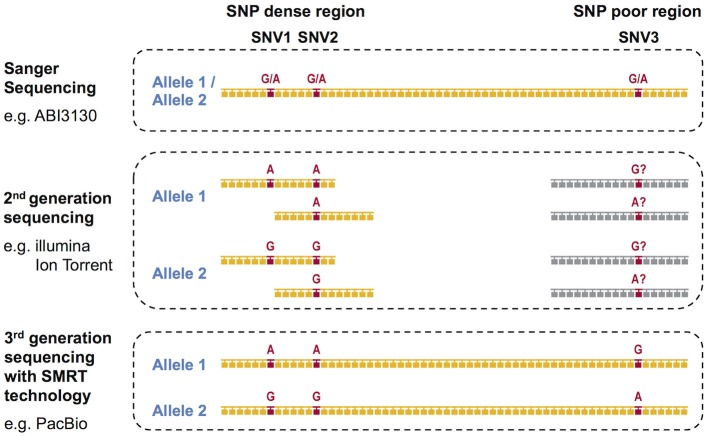
DNA based HLA-genotyping scheme for sequencing platforms representing the first-generation, second-generation and third-generation technologies. The phase ambiguities of the SNV G/A at loci SNV1, SNV2, and SNV3 are easier to resolve using 2nd and/or 3rd generation sequencing than 1st generation Sanger sequencing.

During the past decade, next-generation sequencing (NGS) using mostly second-generation short-read sequencing technologies have employed exon sequencing for some or all of the HLA coding regions in an attempt to solve the phase ambiguity problem encountered by the conventional Sanger Sequencing-Based HLA genotyping methods (23). We previously reported on the development of super high resolution-sequence-based typing application using two NGS platforms, Ion PGM (Life Technologies) and GS Junior (Roche) (24–26). In both of the studies, long-range PCR amplicons from the promoter-enhancer region to 3′UTR for eight classical HLA loci, HLA-A, HLA-B, HLA-C, HLA-DRB1, HLA-DQA1, HLA-DQB1, HLA-DPA1, and HLA-DPB1 were shotgun sequenced. Other long-range PCR and NGS-based shotgun sequencing based HLA genotyping methods using the 454 GS-FLX (Roche) and the MiSeq/HiSeq (Illumina) platforms (18, 27–32) also can resolve many of the phase ambiguities. However, all of these methods are affected by the disadvantages associated with the short-read lengths of NGS technologies, which range from 150 to 500 bp. Although the short-read sequences can accurately separate the two phases in SNP dense regions, such as a polymorphic exon, the phasing is impossible in SNP deficient regions (Figure 1). Specifically, it is necessary to have at least two single nucleotide variants (SNVs) in one short-read sequence to separate both phases. The full-length HLA allele sequences are especially difficult to phase in the HLA-DQA1, HLA-DPA1, and HLA-DPB1 loci because their SNP densities are much less than in the other HLA loci (24), making it unlikely that two SNPs will appear in one short-read sequence.

In contrast to the second-generation sequencers, the third-generation sequencers such as the Single Molecule, Real-Time (SMRT) Sequencing platform PacBio RS II provide continuous long-reads with an average read-length of 10 kb or more. The consensus sequences generated from these long-reads can resolve the limitations of the current second-generation NGS methods as they span most of the long-range PCR amplified regions of the HLA genes [Figure 1, (33, 34)]. Thus, the PacBio platform alone, or in combination with the short-read sequencers such as Ion Torrent and the Illumina platforms, is expected to determine the HLA allele sequences at the full-length level.

In this paper, we describe the generation of a comprehensive collection of full-length HLA allele reference sequences from eight classical HLA loci (HLA-A, HLA-B, HLA-C, HLA-DRB1, HLA-DQA1, HLA-DQB1, HLA-DPA1, and HLA-DPB1) using both the SMRT and the Ion Torrent sequencing technologies. We used 46 genomic DNA samples from the healthy subjects that include 137 high-frequent HLA alleles resolved to the field-2 level (an allele resolution level of sequences that differs by a non-synonymous substitution) in the Japanese population (>99.8% cumulative allele frequencies in each locus). Also, we compared the classified full-length HLA allele sequences at each locus from the perspective of nucleotide diversity of SNVs and insertions or deletions (indels) within the noncoding regions. As an example of future HLA-related disease and transplantation studies, we identified specific variations and previously “undetermined” sequence regions in the HLA-DRB1 locus of these Japanese reference samples that might be related to rheumatoid arthritis (RA).

## Materials and methods

### Genomic DNA samples previously genotyped for HLA polymorphisms to the field-2 level of allelic resolution

A total of 3,115 donors for bone marrow transplantation through the Japan Marrow Donor Program (JMDP) between 2006 and 2010 were retrospectively genotyped by the PCR-SSO-Luminex method for the HLA-A, HLA-B, HLA-C, HLA-DRB1, HLA-DQB1, HLA-DPB1 loci only up to the field-2 level of allelic variant designations as described elsewhere (35). Also, 400 healthy Japanese subjects collected at Tokai University were genotyped by PCR-SSO-Luminex method for the HLA-A, HLA-B, HLA-C, and HLA-DRB1 loci and by the PCR-SBT method for the HLA-DQB1 and HLA-DPB1 loci to the field-2 level (unpublished data). Based on the distribution of the HLA allele frequency data in the Japanese population (HLA laboratory: http://www.hla.or.jp/haplo/haplonavi) (Table S1), a reference set of 46 DNA samples [22 from JMDP (JPN01 to JPN22) and 24 from Tokai University (JPN23 to JPN46)] were selected from the larger number of low-resolution genotyped samples. These Japanese reference samples represented more than 99.6% of the known HLA alleles at each HLA locus excluding HLA-DQA1 and HLA-DPA1 with 137 alleles in total (19 HLA-A, 38 HLA-B, 17 HLA-C, 31 HLA-DRB1, 15 HLA-DQB1, and 17 HLA-DPB1) (Table S1: blue background). The HLA genotyping results limited to the field-2 level of allelic resolution by the traditional HLA genotyping methods are shown in Table S2.

### Long-range PCR

We used the previously designed HLA-A, HLA-B, HLA-C, HLA-DQA1, and HLA-DPA1 locus-specific primer sets that cover the whole gene regions from the promoter-enhancer region to 3′UTR with the expected amplicon size of 5.5 kb for HLA-A, 4.6 kb for HLA-B, and 4.8 kb for HLA-C, 7.5 kb for HLA-DQA1, and 9.7 kb for HLA-DPA1 (24). For each of the HLA-DRB1, HLA-DQB1, and HLA-DPB1 loci the PCR regions were divided into two overlapping parts: 6.1–11.2 kb for the enhancer-promoter to exon 2 (PCR amplicon name: DRB1-1) and 5.5–6.7 kb for exon 2 to 3‘UTR (DRB1-2) in HLA-DRB1; 6.7 kb for the enhancer-promoter to exon 4 (DQB1-1) and 5.7 kb for intron 1–3‘UTR (DQB1-2) in HLA-DQB1; and 5.9 kb for enhancer-promoter to intron 2 (DPB1-1) and 7.3 kb for intron 1–3‘UTR (DPB1-2) in HLA-DPB1 (Figure S1). The PCR conditions were published previously (24). After amplification, the PCR amplicons were purified by the Agencourt AMPure XP (Beckman Coulter, Fullerton, CA) and quantified by the Quant-iT PicoGreen dsDNA Assay Kit (Invitrogen/Thermo Fisher Scientific, Carlsbad, CA) with a Fluoroskan Ascent Microplate Fluorometer (Thermo Fisher Scientific, Waltham, MA). We prepared the PCR amplicons separately for the PacBio and Ion PGM sequencing methods as described in the following sections.

### Sequencing using the PacBio RS II sequencer

We partitioned the 11 long-range PCR amplicons from the eight HLA loci into three groups based on the expected length of the PCR amplicons and constructed three sets of pooled libraries. Pool-1 included HLA-A, -B, and -C (4.6–5.5 kb) gene amplicons, pool-2 consisted of HLA-DRB1-2, -DQB1-1, -DQB1-2, and -DPB1-1 (5.5–6.7 kb) and pool-3 included HLA-DRB1-1, -DQA1, -DPA1, and -DPB1-2 (6.1–11.2 kb) (Figure S1). The quantity and size distribution of the PCR amplicons were measured using the Qubit Fluorometer (Life Technologies/Thermo Fisher Scientific, Palo Alto, CA) and Agilent 2100 Bioanalyzer DNA12000 kit (Agilent Technologies, Santa Clara, CA). The total amount of pooled amplicon DNA input into each library preparation ranged between 3 and 4 micrograms. Using the PacBio DNA Template Prep Kit 2.0 and SMRTbell Barcoded Adapter Complete Prep Kit (Pacific Biosciences, Menlo Park, CA), the pooled PCR amplicons were end-repaired, and blunt-end ligated to barcoded hairpin adapters to produce SMRTbell templates. A 0.45 × volume of AMPure PB beads was used to purify the barcoded SMRTbell libraries before sequencing. A binding calculator provided by PacBio determined the ratio of sequencing primer to template and polymerase to template and optimal loading concentration in the SMRT Cell. The SMRTbell templates were bound to P6 DNA polymerase. To remove excess polymerase and primer, the SMRTbell-polymerase complex was bound to MagBeads at 4°C for 20 min, using manufacturer's guidelines. The MagBeads-bound polymerase-template complexes were run in the PacBio RS II for sequencing. The most advanced sequencing chemistry (P6-C4) for the PacBio RSII Sequencing System, was used to generate long-read sequences spanning the complete lengths of the input long-range PCR amplicons. The most optimal movie collection time was employed to capture full-length reads. A 240-min movie was collected for the first pool (4.6–5.5 kb), and 360 min movies were collected for the second (5.5–6.7 kb), and third (6.1–11.2 kb) libraries to produce maximum number of full-length reads. In total, six SMRT Cells were run for sequencing, two for each of the pooled libraries 1, 2, and 3. After sequencing, the analysis of the consensus sequences was done independently for each of the pooled libraries 1, 2, and 3. Clustering, phasing, consensus building, post-processing and evaluation of the consensus sequences were performed using the Long Amplicon Analysis (LAA) computing pipeline of the SMRT Analysis v2.3 program (http://www.pacb.com/wp-content/uploads/guide-pacbio-HLA-getting-started.pdf). Sequencing data were first filtered to remove all subreads with predicted accuracy of <80%, lengths of <90% of the shortest amplicon in each pool, and a signal-to-noise ratio (SNR) of less than 4.0. For each pool, up to 5,000 of the remaining high-quality, full-length reads were used for the LAA analysis with default phasing parameters (maximum phasing reads = 500, minimum split fraction = 0.1).

### Sequencing using the ion PGM sequencer

Barcoded-library DNA samples were prepared with an Ion Xpress Plus Fragment Library Kit and Ion Xpress barcode Adaptors 1–96 Kit according to the manufacturer's protocol for 400 base-read sequencing (Life Technologies/Thermo Fisher Scientific). One hundred nanograms of the multiplex PCR amplicons were used for the preparation of each DNA barcoded-library. DNA samples were fragmented with an M220 Focused-ultrasonicator (Covaris, Woburn, MA). Eight cycles of PCR were used to amplify each DNA library. The DNA size and quantitation for each library were measured with an Agilent 2100 Expert Bioanalyzer using the Agilent High Sensitivity DNA Kit (Agilent Technologies). Each barcoded-library was mixed at equimolar concentrations then diluted according to the manufacturer's recommendation. Emulsion PCR (emPCR) was performed on the resulting pooled library with the Ion PGM Template OT2 400 Kit on an Ion OneTouch 2 automated system (Life Technologies/Thermo Fisher Scientific). After the emulsion was automatically broken with the OneTouch 2 instrument, the beads carrying the single-stranded DNA templates were enriched according to the manufacturer's recommendation. Sequencing was performed using the Ion PGM Sequencing 400 Kit and Ion 318 Chip Kit v2 with a flow number of 850 for a 400 base-read (36). The raw data processing and base-calling, trimming, and output of quality-filter sequence reads were binned by the Ion Xpress Barcodes into 46 separate sequence fastq files using the Torrent Suite 4.2.1 software (Life Technologies/Thermo Fisher Scientific) with full processing for shotgun analysis. These sequence data files were further quality trimmed to remove poor sequences at the end of the reads with Quality values (QVs) of <15. The trimmed and barcode-binned sequence reads were used for HLA allele assignment to the field-2 level using the in-house Sequence Alignment Based Assigning Software [SeaBass, Tokai University, (37)]. QVs of sequence reads were calculated by the fastx_quality_stats included with the FASTX-Toolkit for short-reads data preprocessing (http://hannonlab.cshl.edu/fastx_toolkit/).

### Allele sequence determination of full-length reference sequences

We used two complementing sequencing technologies to characterize the HLA alleles; generally, the PGM short-read sequences for genotyping and the PacBio long-read consensus sequences to phase the HLA allele sequences. In order to obtain more accurate nucleotide sequences, short-read sequence outputs by Ion PGM sequencing were mapped to the long-read consensus sequences that were produced by PacBio RS II sequencing using the GS Reference Mapper Ver. 3.0 software (Roche, Basel, Switzerland). To avoid mismapping among the HLA loci and contamination of the *in vitro* generated PCR crossover amplicons (37, 38), we first used the software mapping parameters that found perfect alignment matches between the read sequences and the reference sequences using the following settings: Minimum overlap identity: 100, Minimum read length: 45, Minimum overlap length: 200, Alignment identity score: 10, and Alignment difference score: 0. We confirmed high percent coverage of the references in the Profile window, and visually inspected the sequence alignments for nucleotide mismatches in the results window using the parameter setting called Open the Alignment. The long-read consensus sequences were considered to be correct when they and the short-read sequences aligned uniformly and correctly to the reference sequence. When there were unmapped nucleotide sites or mismatches in the sequence alignments, we realigned the short-read sequences to the long-read consensus sequences by changing the Minimum overlap identity parameter from 100 to 99. From the new alignment information obtained at a slightly lower stringency, we manually modified the long-read consensus sequences based on the information of direct matches between the short-read sequences and the previously unmodified consensus sequences. The modified long-read consensus sequences that remained doubtful were checked and confirmed by Sanger direct-sequencing.

### Sanger sequencing using the ABI3130xl genetic analyzer

Purified PCR amplicons were sequenced bidirectionally, to validate newly discovered SNVs and indels with the Big Dye Terminator Kit v.1.1 (Life Technologies/Thermo Fisher Scientific) using the ABI3130xl genetic analyzer (Life Technologies/Thermo Fisher Scientific). The generated chromatogram sequence data were analyzed by the Sequencher ver.5.0.1 DNA sequence assembly software (GENCODE).

### Nucleotide diversity analysis

Multiple sequence alignments for each locus were created using the Sequencher ver.5.0.1 DNA sequence assembly software (GENCODE). The diversity profile was calculated and drawn using the graphics output of the Microsoft Excel for Mac 2011. Repetitive elements including retroelements were identified within the HLA allele sequences by using the RepeatMasker webserver (http://www.repeatmasker.org).

### Phylogenetic analysis

Multiple sequence alignments for each locus were created using the ClustalW Sequence Alignment program of the Molecular Evolution Genetics Analysis software 6 (MEGA6) (39). Phylogenetic trees were constructed by the Neighbor-joining (NJ) method with Maximum Composite Likelihood model and assessed using 10,000 bootstrap replicates in the MEGA6 software (40).

### Allele assignments and nomenclature

The HLA alleles were assigned using HLA Fusion 3.0 Software (One Lambda/Life Technologies, Canoga Park CA) for the PCR-SSO-Luminex data, u-Type 7.1 software (One Lambda) for the SBT data, and Blat sequence alignment search for the downloaded IPD-IMGT/HLA alleles (Release 3.31.0) and for the PacBio and Ion PGM data. The IPD-IMGT/HLA database assigned the HLA allele official names following the nomenclature for factors of the HLA System using the four-fields of allelic resolution (allelic variants) based on the different levels of variation in the DNA sequences. (41). Field-1 is the allelic lineage or group that is shared by all allele variants in that group; field-2 defines a DNA variation that leads to an amino acid difference (non-synonymous DNA substitution) in the allele group defined by field-1; field-3 indicates the synonymous DNA substitution in the coding region of the allelic group; and field-4 refers to differences in the non-coding regions of the allelic group. (42). HLA exonic sequencing is limited to the field-3 level of allelic resolution, whereas HLA genomic coding and non-coding sequencing extends the allelic resolution up to the field-4 level. However, a unique allele with no variants that has been completely sequenced at the genomic field-4 level is usually given only a two-field name (such as B^*^15:428), and although somewhat confusing, we have retained this practice here.

## Results

### Characteristics of draft-reads for PacBio RS II and ion PGM sequencing

Raw subread information of draft-reads obtained for 46 genomic DNA samples after sequencing the 11 long-range PCR amplicons by the PacBio RS II sequencer is shown in Table S3 and includes SMRT Cell numbers, P1 and P2 values that mean the rates of productive ZMW and other ZMW in SMRT cells, respectively, the minimum, mean, and maximum subread numbers and mean quality values per sample for each primer set. The mean P1-values ranged from 36.7 to 46.1% and the mean P2-values ranged from 6.7 to 10.1%. The highest and lowest mean numbers were observed for the DQB1-1+DQB1-2 primer sets (1,319 subreads ranging from 567 to 2,071) and the DPA1 primer set (63 subreads ranging from 44 to 82), respectively. Less than one hundred minimum subreads were obtained in pool-3 that included long-range PCR amplicons of 6.1–11.2 kb, whereas over one hundred minimum subreads were obtained in pool-1 and pool-2 that included comparatively shorter PCR amplicons of 4.6–6.7 kb. No subreads were obtained for allele 2 of the DPA1 gene in JPN29. In total, 692 consensus sequences were generated from the subreads using the LAA pipeline in preparation for constructing and mapping the full-length HLA allele sequences using the consensus sequences.

A summary of the number of draft-read bases obtained from 46 genomic DNA samples after sequencing individual HLA loci using the Ion PGM sequencer is presented in Table S4. Draft-read numbers were high-quality reads, and in total there was 15,016,797 sequence reads from two runs with read numbers ranging from 106,840 in JPN10 to 707,242 in JPN33 [326,452.1 ± 141,566.9 standard deviation (SD) on average]. The average quality score and SD were 27.0 ± 0.6. The draft-read bases in total were 3.8 Gb with a range between 23.1 Mb in JPN10 and 159.1 Mb in JPN33 (83.2 ± 35.3 Mb on average), with an overall average, read length of 255.9 ± 12.8 bases.

### Comparison between the PacBio and ion PGM data for coding sequences

From a nucleotide similarity search of short-read sequences by Ion PGM sequencing 44 homozygous genotypes were identified from a total of 368 genotyped (46 samples × 8 loci) coding sequences (CDSs) (Table S5). Allele dropouts were not included in the analyses of these HLA-A, -B, -C, -DRB1, -DQB1, and -DPB1 sequences because the genotypes were consistent with the results of PCR-SSO and SBT (Table S1). However, it is possible that there was allele dropout in the homozygous genotypes of HLA-DPA1 and -DQA1. The five alleles represented by the HLA-B genes of JPN34 and JPN36, and HLA-DPA1 of JPN04, JPN25, and JPN37 showed 99.9 and 99.4% nucleotide similarity to the most similar previously documented alleles, respectively, while the other alleles were perfectly matched to known sequences. All typing data were identical with the retrospectively genotyped data (Table S2), excluding a single discrepancy for HLA-C^*^08:01 in JPN04 and HLA-C^*^08:22 in JPN30 due to an SNV that separated the alleles in exon 6.

The PacBio consensus sequences were used as reference sequences for mapping the short-read sequence outputs by Ion PGM sequencing. Although the output from the PacBio RS II sequencing identified 44 homozygous genotypes, the comparison of the nucleotide similarities between both sets of sequencing data revealed artifactual dropouts for four alleles, HLA-B of JPN04, HLA-DQA1 of JPN10, and HLA-DPA1 of JPN29, and a mismatch for one allele of HLA-DQB1 of JPN20 (Table S5). DNA typing of these samples by the PCR-SSO-Luminex and PCR-SBT methods confirmed that these were dropouts in the PacBio data. The four dropout allele sequences were determined by comparing the most similar allele sequences within the reference sequence group against those that were determined in other samples using the Ion PGM reads.

### Characterization and allele assignment of the full-length, polymorphic class I and class II HLA sequences to establish a population reference group

A total of 253 HLA allele sequences (20 HLA-A, 46 HLA-B, 26 HLA-C, 41 HLA-DRB1, 36 HLA-DQA1, 28 HLA-DQB1, 16 HLA-DPA1, and 40 HLA-DPB1) that covered the promoter-enhancer region, the intronic and exonic regions and across to 3′UTR were determined for the 46 Japanese samples by the PacBio, Ion PGM, and Sanger sequencing. Table S6 shows the genomic allele designation of the full-length HLA alleles (coding and non-coding sequences) up to the field-4 level for some variants for each sample and locus. Table S7 shows the allele names, DNA data bank of Japan (DDBJ) accession numbers, IMGT submission numbers, nucleotide length of each allele, novel or extended alleles, the location of variations, and the number of detected alleles.

The two alleles B^*^40:01:02:01/04 and DQB1^*^03:03:02:02/03 were classified as ambiguous because some nucleotide variations previously used to assign these alleles were located outside the region of our two sequences. Of the 736 alleles, 181 alleles (24.6%) were novel, and 23.9% (176/736) of them had variations within introns or in the 5′ and 3′ non-coding regions. Also, 193 alleles were extended from an exonic or partial gene sequence to a full-gene sequence, and 50.8% of the 736 alleles were reassigned in the IPD-IMGT/HLA database as novel allele sequences. Also, 89.6% of the HLA-DRB1 allele sequences are unique or extended alleles (Table 1 and Table S7). Of the 101 novel alleles, four had variants in the exonic regions, and of them, B^*^15:428, DPA1^*^02:07:01:01, and DPA1^*^02:08 had non-synonymous substitutions (Table 2). The five alleles (HLA-B of JPN34 and JPN36 and HLA-DPA1 of JPN04, JPN25, and JPN37) that showed 99.9 or 99.4% nucleotide similarities in the CDS search were also classified as novel alleles (Table S5). Of the 44 genotypes that initially were classified as homozygous within the coding regions, seven of them were revealed as heterozygotes within the intronic region (Table S5).

**Table 1:** Characterization of 253 distinct full-length HLA alleles.

**Locus**	**Distinct sequences**	**Total sequences**	**Detected novel allele number**			**Novel alleles/all alleles**	**Detected extended allele number**	**Novel + extended alleles/all alleles**
			**Coding region**	**Intronic region**	**5′/3′Non-coding region**			
A	20	92	0	1	0	1.1%	2	3.3%
B	46	92	2	5	7	15.2%	8	23.9%
C	26	92	0	2	9	12.0%	29	43.5%
DRB1	41	92	0	45	0	48.9%	37	89.1%
DQA1	36	92	0	28	1	31.5%	21	54.3%
DQB1	28	92	0	32	5	40.2%	5	45.7%
DPA1	16	92	3	10	1	15.2%	69	90.2%
DPB1	40	92	0	30	0	32.6%	22	56.5%
Total	253	736	5	153	23	24.6%	193	50.8%

**Table 2:** Identification and classification of novel variants in exonic regions.

**Sample ID**	**JPN34**	**JPN36**	**JPN04, JPN25**			**JPN37**
Locus	HLA-B	HLA-B	HLA-DPA1			HLA-DPA1
Novel allele name	B*15:428	B*39:02:03	DPA1*02:07:01:01			DPA1*02:08
Reference	B*15:28	B*39:02:02	DPA1*02:02:01			DPA1*02:01:01:02
IMGT num. of reference	HLA00191	HLA00275	HLA00508			HLA14197
Reference position	25	1,008	251	361	442	4893
Location	Exon 1	Exon 5	Exon 3	Exon 3	Exon 3	Exon 4
Nucleotide Reference	G	C	G	A	A	G
Nucleotide Variant	C	T	A	G	G	A
Amino acid substitution	V9L	-	V122M	-	-	R249H

*A hyphen indicates synonymous substitution*.

### Nucleotide diversity and phylogenetic analyses of HLA allele sequences

To calculate and map the nucleotide diversities for each HLA locus we constructed multiple sequence alignments and nucleotide diversity profiles at the locus level using all of the allele sequences identified in this study (Figure S2). The indel numbers and SNV rates (SNV/Kb: number of SNVs/alignment length excluding indels/allele numbers × 1000) in the class I loci (HLA-A, HLA-B, and HLA-C) showed comparatively low rates with 11–13 numbers for indels and 1.4–3.1 SNV/Kb (Table 3). The indels were dispersed mainly within the intronic regions, whereas highest SNV/Kb were observed in the exonic regions such as exons 2 and 3 in HLA-A and HLA-B and across all the exons in HLA-C (Figures S2A–C). In comparison to the class I loci, the class II loci showed a higher level of locus-specific indels with 22–144 numbers and a moderately high level of SNV rates with 2.2–5.3/Kb at the allele level (Table 3).

**Table 3:** The number of indels and SNVs among the alleles at each HLA locus.

**Locus**	**Allele number to the full-length level**	**Including indels**		**Excluding indels**		**SNV/Kb**
		**Alignment length (bp)**	**Number of indels**	**Alignment length (bp)**	**Number of SNVs**	
A	20	5,436	13	5,396	336	3.1
B	46	4,575	11	4,541	285	1.4
C	26	4,760	12	4,733	246	2.0
DRB1	41	18,740	144	10,262	1,727	2.2
DQA1	36	7,801	77	7,240	957	3.4
DQB1	28	8,393	107	7,551	1,247	5.3
DPA1	16	9,766	28	9,662	348	2.2
DPB1	40	12,306	22	12,147	343	0.7

**Figure 2:**
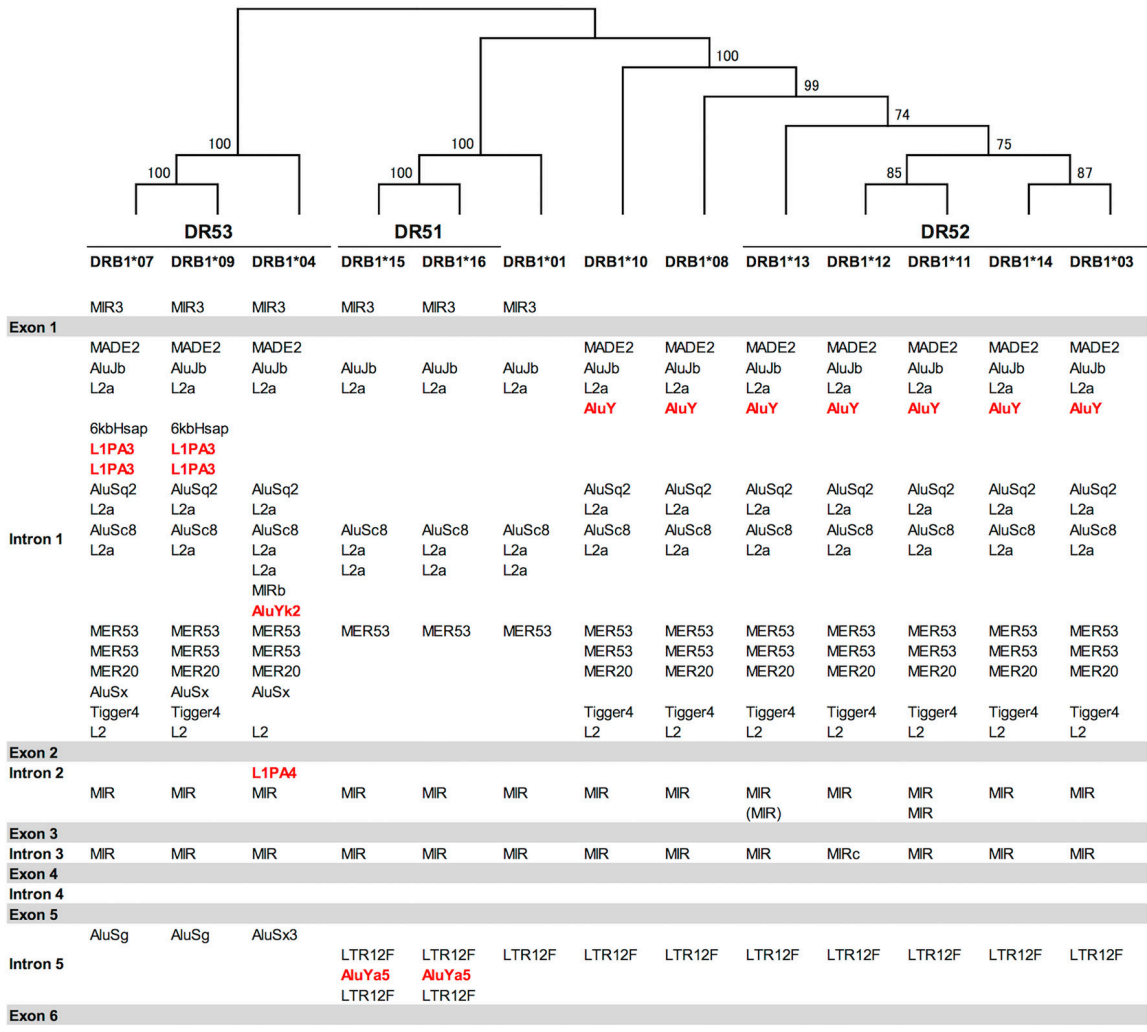
Gene structure, DR supratypes and phylogenetic relationships using the HLA-DRB1 allele sequences. Three of the DR supratypes are labeled DR53, DR51, and DR52, and their component alleles are listed underneath the horizontal lines. The phylogenetic tree of the HLA-DRB1 alleles ranging from DRB1^*^07 to the left of the figure and DRB1^*^03 to the right of the figure was constructed using the Neighbor-Joining method and a 1,962 bp nucleotide alignment that included 261 bp of AluJb, 492 bp of L2a, 281 bp of AluSc8, 655 bp of L2a, and 65 bp of MER53 in intron 1, and 145 bp of MIR in intron 2 and 63 bp of MIR in intron 5. Numbers on the branches are bootstrap support values. Red letters indicate Alu and LINE sequences that may have been inserted in the comparatively recent period during the last 10 million years. Gray bars indicate the exonic regions and the white open regions with the retroelement lists between the gray bars represent introns 1 to 5 in between exons 1 and 6.

Indels of over 500 bp were observed in introns 1, 2, and 5, and many of them explicitly correlated with the DR supratypes. Most of the indels were composed of repetitive elements such as short interspersed nuclear element (SINE) and long interspersed nuclear element (LINE) sequences located in position 5,264–10,528 bp of intron 1, position 2,214–3,464 bp of intron 2 and position 627–1,142 bp of intron 5 (Figure 2 and Figure S2D). This result of the correlation between structurally polymorphic retrotransposons and the HLA-DR supratypes DR1, DR8, DR51, DR52, and DR53 supports and extends the previous findings (43, 44). The relationships of the DR supratypes and the indels also were strongly supported by a phylogenetic tree that was constructed using selected intronic 1, 2, and 5 sequences (Figure 2). Also, the transcription factor binding motifs such as X1, X2, Y, CCAAT, and TATA boxes (45) appear to have evolved according to the lineages of the DR-types (data not shown). The highest SNV/Kb were observed mostly around the exon 2 and intron 5 regions.

In regard to the nucleotide diversity within the other class II genes, the indel numbers were 77 and 107 for the HLA-DQA1 and HLA-DQB1 loci, respectively, and significant indels were observed in the introns 2 and 3 of HLA-DQA1 and intron 2 of HLA-DQB1 (Figures S2E,F). The SNV/Kb was 3.4 and 5.3 for HLA-DQA1 and HLA-DQB1, respectively, which was similar for the HLA-DRB1 locus (Table 3). The highest SNV/Kb were observed around the 5′UTR, exon 2 and exon 5 regions in HLA-DQA1 and around exon 2 within HLA-DQB1. The HLA-DPA1 and HLA-DPB1 loci were well conserved among the class II genes with 28 and 22 indel numbers and 2.2 and 0.7 SNV/Kb, respectively (Table 3). The 250 bp up-stream regions of the loci that include the transcription factor binding motifs were perfectly conserved among the alleles, although allelic variations in the transcription factor binding motifs were observed within some of the other HLA loci (data not shown).

**Figure 3:**
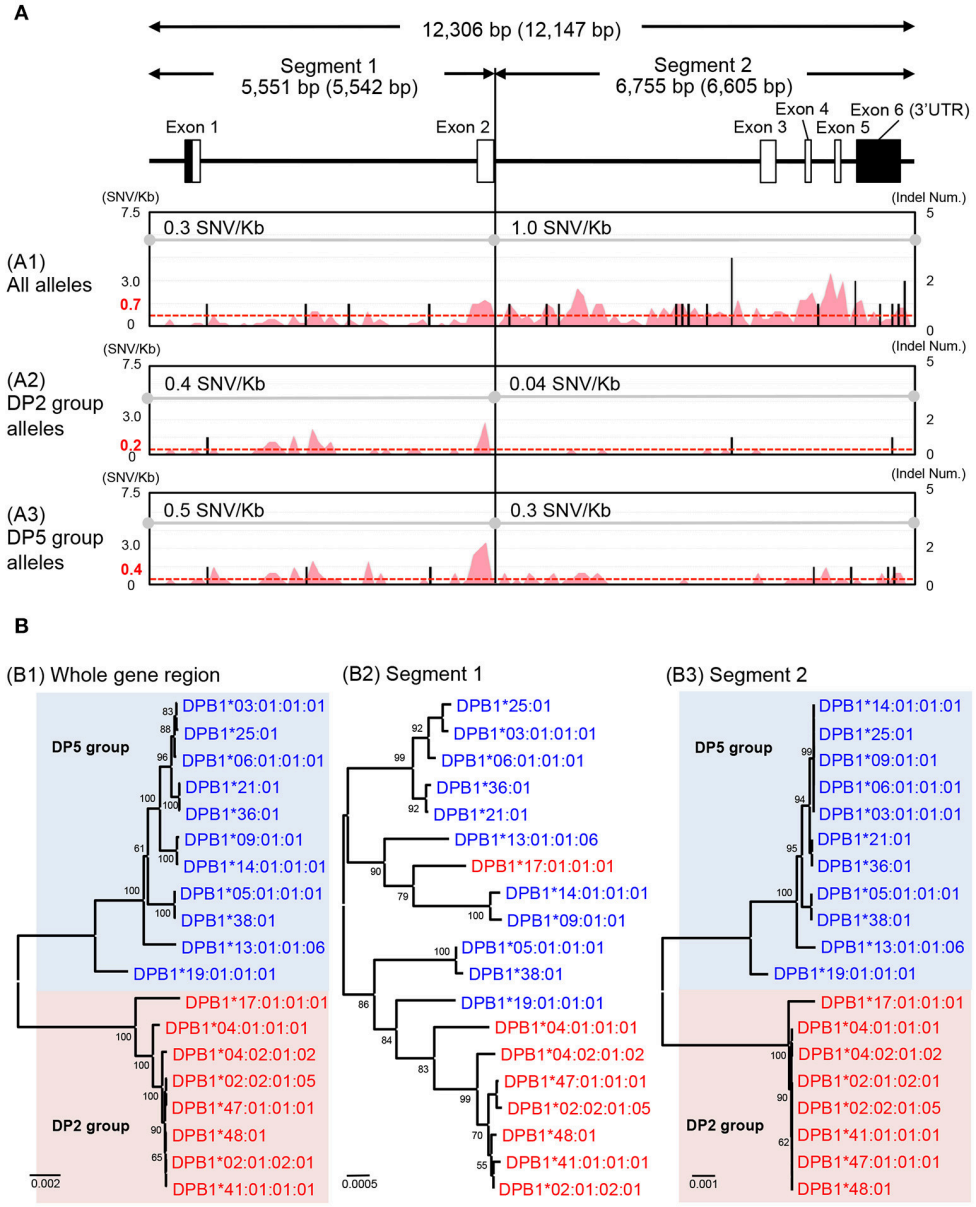
Nucleotide diversity and phylogenetic analyses using the HLA-DPB1 allele sequences. **(A)** Nucleotide diversity profiles were constructed using three nucleotide alignments, 12,306 bp (whole gene region), 5,551 bp (segment 1), and 6,755 bp (segment 2). The nucleotide lengths (bp) are shown with indels (no parenthesis) and without indels (in parenthesis). The A1, A2, and A3 matrix windows show the diversity profiles using 38 HLA-DPB1 alleles (18 DP2 group alleles and 20 DP5 group alleles), except for two recombinant alleles, DPB1^*^17:01:01:01 and DPB1^*^19:01:01:01. The red peak and valley profiles within the matrix windows indicate the changes in SNV/Kb across the aligned sequences and the black bars indicate indel numbers among the alleles. The average SNV/Kb for the sequence alignment is shown on the top line of each matrix window. **(B)** Phylogenetic trees using the 19 representative HLA-DPB1 alleles were constructed by the Neighbor-Joining method. The three phylogenetic trees (B1–B3) represent the 12,147 bp nucleotide alignment of HLA-DPB1 whole gene region (B1), the 5,542 bp nucleotide alignment of the enhancer-promoter region to exon 2 (B2), and the 6,605 bp nucleotide alignment of the intron 2–3′UTR region (B3). Red letters and backgrounds indicate the DP2 group (rs9277534: A) and blue letters and backgrounds indicate the DP5 group (rs9277534: G) alleles. Numbers on the branches are bootstrap support values.

The HLA-DPA1 and HLA-DPB1 allele sequences were separated into two distinct divergent groups, the DP2 and DP5 (46). The nucleotide diversity profiles of the HLA-DPA1 (Figure S2G) and HLA-DPB1 (Figure 3A1) showed unusually low diversity patterns when compared to the other HLA class II loci (Table 3 and Figures S2D–F). Although the diversity patterns of the different HLA class II loci revealed the presence of a high SNV/Kb throughout the gene regions (2.2–5.3/Kb) and high SNV densities in the polymorphic exons (Figures S2D–F), the HLA-DPB1 nucleotide diversity profiles of the DP2 and DP5 groups included SNV/Kb of 0.3 for the 5.5 kb region between the promoter-enhancer and exon 2 (Segment 1) and 1.0 for the 6.6 kb region between intron 2 and the 3′UTR (Segment 2) (Figure 3A1).

The HLA-DPB1 variation at rs9277534 has been associated with HLA-DPB1 expression levels, whereby high expression was associated with rs9277534G and low expression was associated with rs9277534A (20). Because the rs9277534 site was located in the 3′UTR region of our HLA-DPB1 allele sequences, we grouped the rs9277534G with DP5 and rs9277534A with DP2, excluding two recombinant alleles, DPB1^*^17:01:01:01 and DPB1^*^19:01:01:01. By comparing various allelic sequences, we deduced that DPB1^*^17:01:01:01 in the DP2 group and DPB1^*^19:01:01:01 in the DP5 group were generated by recombination events in intron 2 of the DPB1^*^02:01:02:01 and DPB1^*^05:01:01:01 alleles (Figure S3).

We also compared the nucleotide diversity of the DP2 against DP5 and found that the SNV/Kb were markedly reduced in both the DP2 and DP5 groups with 0.4 and 0.5 in segment 1 and 0.04 and 0.3 in the segment 2, respectively (Figures 3A2,A3). The SNV/Kb in both of these segments were lower than those for all the alleles across the whole gene region (Figure 3A1). However, relatively high diversity was maintained within exon 2 of both DP groups, and the polymorphisms of exon 2 appear to have evolved independently of the DP2 and DP5 structures. This hypothesis is supported by the phylogenetic analyses of the HLA-DPB1 allele sequences (Figure 3B), whereby the trees for Segments 1 and 2 (Figures 3B2,B3) showed similar phylogenies, excluding the recombinant alleles, and that the tree for Segment 2 (Figure 3B3) clearly separated into the DP2 and DP5 groups. However, most of the branches of Segment 1 are longer than those of Segment 2, which is consistent with the view that most of the polymorphisms or variations were generated in Segment 1.

### Identification of novel SNVs and sequence region variations in DRB1^*^04 allele sequences previously associated with rheumatoid arthritis (RA)

We extended the sequences for four DRB1^*^04 alleles and identified novel intronic variants for five DRB1^*^04 alleles (Table S7) that could be separated into three rheumatoid arthritis (RA) categories based on the previous results of a case-control study using 1480 patients and 800 healthy controls in the Japanese population (47). All together, these were RA-susceptible alleles for DRB1^*^04:01:01:03, DRB1^*^04:05:01:01, DRB1^*^04:05:01:02, DRB1^*^04:05:01:03, and DRB1^*^04:10:03, RA-resistant alleles for DRB1^*^04:03:01:02, DRB1^*^04:06:01, and DRB1^*^04:07:01:02, and a RA-non-associated allele for DRB1^*^04:04:01. Although it has been previously shown that amino acid 74 in exon 2 (nucleotide position 8,563) correlates well with RA susceptibility, the extended intronic sequence information enabled us to identify in addition two positions in intron 2 correlating with RA susceptibility as well at nucleotide positions 9,139 and 9,304 of the sequence alignments using the DRB1^*^04 group sequences in the IPD-IMGT/HLA database after repeat sequence masking (Figure S4 in Supplementary Material). The “A” allele at the position 9,304 was observed in the RA-susceptible alleles (DRB1^*^04:01:01:03, DRB1^*^04:05:01:01, DRB1^*^04:05:01:02, DRB1^*^04:05:01:03, and DRB1^*^04:10:03). The “G” allele was observed in the RA-resistant alleles (DRB1^*^04:03:01:02, DRB1^*^04:06:01, and DRB1^*^04:07:01:02). Interestingly, a deletion of two bases was observed in the other 32 HLA-DRB1 alleles. In addition, a microRNA (hsa-miR-7156-5p) was predicted to bind to the RA resistant allele sequences at the position 9,304, but not to the other allele sequences when we examined the miRbase (http://www.mirbase.org) using a microRNA target prediction software (Figure S4). In contrast to the allele at the position 9,304, the “A” allele at position 9,139 was observed in the RA-resistant alleles (DRB1^*^04:03:01:02, DRB1^*^04:06:01, and DRB1^*^04:07:01:02) and RA-non-association allele (DRB1^*^04:04:01), whereas the “G” allele was observed in the other 37 HLA-DRB1 alleles including the RA-susceptible alleles (Table 4). Furthermore, Table 4 shows that while the presence of the polymorphic AluYK2 and L1PA4 retroelements (Figure 2) correlated with all the DRB1^*^04, the SNV at 9,139 and 9,304 differentiated between resistant and susceptible RA forms.

**Table 4:** Comparison of rheumatoid arthritis (RA)-susceptible, resistant and non-association alleles in the DRB1*04 group sequences.

**Statistical information of Oka et al. [****47****]**			**Allele name at the full-length level**	**Amino acid position in exon 2**								**Nucleotide position in intron 2**		**Presence of AluYk2 in intron 1**	**Presence of L1PA4 in intron 1**
**Allele name**	***P*-value**	**OR (95% CI)**		**67**	**68**	**69**	**70**	**71**	**72**	**73**	**74**	**9139**	**9304**		
DRB1*04:05	1.4 × 10e-36	3.3 (2.73–4.01)	DRB1*04:05:01:01	L	L	E	Q	R	R	A	**A**	**G**	**A**	(+)	(+)
			DRB1*04:05:01:02											(+)	(+)
			DRB1*04:05:01:03											(+)	(+)
DRB1*04:01	4.3 × 10e-5	2.8 (1.63–4.70)	DRB1*04:01:01:03	L	L	E	Q	K	R	A	**A**	**G**	**A**	(+)	(+)
DRB1*04:10	0.01	1.8 (1.12–3.02)	DRB1*04:10:03	L	L	E	Q	R	R	A	**A**	**G**	**A**	(+)	(+)
DRB1*04:06	0.0005	0.5 (0.35–0.74)	DRB1*04:06:01	L	L	E	Q	R	R	A	**E**	**A**	**G**	(+)	(+)
DRB1*04:03	0.0012	0.5 (0.30–0.74)	DRB1*04:03:01:02	L	L	E	Q	R	R	A	**E**	**A**	**G**	(+)	(+)
DRB1*04:07	0.0001	0.1 (0.05–0.43)	DRB1^*^04:07:01:02	L	L	E	Q	R	R	A	**E**	**A**	**G**	(+)	(+)
Other alleles				LIF	L	E	QRD	RKEA	R	AG	AEQLR	**G**	–	–	–

RA-susceptible (red) and resistant (blue) alleles are shown in this table. Amino acid and nucleotide positions are bases on the sequence alignment tool of the IPD-IMGT/HLA database (https://www.ebi.ac.uk/ipd/imgt/hla/align.html) using the DRB1^*^04 group sequences shown in Figure S4 in Supplementary Material. The Bold letter indicates a difference of the amino acid or nucleotide sequence between the RA susceptible and resistant alleles. Amino acid positions in the exon 2 were referred from (47–51).

## Discussion

In this study, a total of 253 full-length HLA allele sequences were determined from 46 healthy subjects with a cumulative frequency of more than 99.2% (ranging from HLA-DQA1: 99.2% to HLA-DQB1: 100%) in the Japanese population (Table S1). Of this collection of reference sequences, 50.8% were characterized to be novel and extended alleles (Table 1), and of these, 67.2% (309 alleles/460 alleles) of class II alleles were novel or extended. Only three of the novel HLA alleles were non-synonymous substitutions and they were within exon 1 of B^*^15:428, exon 3 of DPA1^*^02:07:01:01, and exon 4 of DPA1^*^02:08 (Table 2). While we focused in this study on producing a full-length HLA reference set for the Japanese population, others have used the PacBio sequencing technologies to determine the full-length HLA allele sequences at six classical loci for 126 International HLA and Immunogenetics Workshop cell lines (52) and the full-length characterisation of 1056 HLA alleles (53). Together, these two-separate long-range PacBio sequencing studies have provided a new set of fully characterized full-length HLA allele reference sequences for future large-scale analyses that can be expected to yield additional novel alleles and lead to more accurate HLA genotyping using cheaper and better conventional methods and strategies.

We used both the PacBio and the Ion PGM sequencing technologies to determine the full-length HLA allele genomic sequences that now represent our collection of Japanese reference alleles for coding and non-coding sequences assigned up to the field-4 level to improve future use in routine genotyping. HLA alleles can only be resolved to the field-4 level if the entire gene is sequenced, whereas exonic sequencing is limited to only the field-2 or field-3 level and not beyond. However, unique alleles that have no variants and have been fully sequenced (exons and non-coding regions) also may be identified only to the field-2 level of resolution (such as B^*^15:428 identified in this study) because the field-3 and field-4 identifiers are omitted from the 4-field identification code of allelic resolution. Therefore, the use of the official HLA nomenclature to only the field-2 level of allelic resolution can be confusing because it might imply that either the allele is unique with no sequence variants or that the allele has been sequenced only to the exonic level and that it has not included any of the non-coding sequences. Although it is normally understood that a unique and fully sequenced HLA allele with no sequence variation will be identified only to the 2-level of resolution without the need to add on the field-3 or field-4 identifiers (e.g., B^*^15:428), it might be better to also add them even to the unique alleles in order to avoid confusion, (e.g., B^*^15:428:0:0). The usual omission of the field- 3 and field-4 identifiers from some alleles may need to be changed in the future to distinguish between the fully sequenced unique alleles and the partially exonic-sequenced genes with no non-coding sequence data (e.g., B^*^15:428:-: -). If there are one or more allelic variants with differences in the non-coding sequences then by definition these allelic variants will be identified to the field-4 level of resolution by also including the field-1, field-2, and field-3 identifiers (e.g., B^*^15:428:0:1).

In comparing, the PacBio and the Ion PGM sequencing technologies, we found both methods had their particular limitations with allele misreads and “drop-outs” and problems with homopolymers, four “drop-out” alleles and one mismatched allele were detected in the PacBio data after a comprehensive nucleotide similarity search (Table S5). These “artifactual” alleles were likely the result of using low-quality nucleotide subreads during the construction of the consensus sequences. The concordance rate between the PacBio consensus sequences and the finally determined nucleotide sequences (concordance rate = sum of correct allele length (bp) from the PacBio consensus sequences /sum of all allele length (bp) from the finally determined nucleotide sequences × 100) was 99.985% for the eight HLA loci with the mismatched alleles. The concordance rates for the class I loci composed of comparatively short PCR amplicons and the class II loci consisting of relatively longer PCR amplicons were 99.999 and 99.987%, respectively. The largest nucleotide differences were observed for HLA-DPA1 (99.976% similarity) and HLA-DRB1 (99.976% similarity) possibly due to the low amount of DNA used for the PacBio library construction of HLA-DPA1 and the resultant output of subreads of specific HLA-DRB1 alleles that had allelic imbalances generated by the long-range PCR. On the other hand, the concordance rate was 100% in the HLA-C locus for which the mean and minimum subreads per sample were 554 and 424, respectively (Table S3). In previous studies of HLA genotyping by second-generation NGS, we found that the determination of the full-length HLA allele sequences was difficult in the HLA-DQA1, HLA-DPA1, and HLA-DPB1 loci because the SNPs and indel densities that were used to separate the chromosomal phases were much lower than in the other HLA loci (24). However, construction of the consensus sequences for the three HLA class II loci using PacBio sequencing was extremely helpful for improving the characterization and classification of HLA allele sequences from the short-reads generated by Ion Torrent and Sanger sequencing. In this regard, PacBio sequencing is a helpful tool for providing reliable and accurate HLA allele sequences if enough high-quality subreads (minimum subread number of more than 300 and average subread number of more than 500) can be provided for the construction of the consensus sequences.

We also encountered problems with some microsatellite and mono-stretch sequences using both technologies, especially for HLA-DRB1 with the complexity of microsatellite polymorphisms in intron 2. Because sequencing errors due to the presence of microsatellite polymorphisms and mono-stretch sequences, either *in vivo* or generated *in vitro* by PCR, will be observed frequently in future, the problematic sequencing sites should be included in the HLA allele definitions such as DRB1^*^15:01:01:01/02/03.

Overall, we obtained highly accurate HLA allele long-read sequences in most of the PCR regions using PacBio SMRT sequencing without a need to confirm the alleles by also using second-generation sequencing. We could have checked some ambiguous nucleotides and newly discovered variations by using Sanger sequencing instead of the second-generation sequencing method. However, the running cost for PacBio SMRT sequencing is collectively higher than for second-generation sequencing.

Although peptide presentation preferences are the key factor influencing disease susceptibility [(47) and Table 4], other factors such as miRNA sequence variants within the coding and noncoding regions are emerging as potential and previously unknown influencing factors (54). The previous limitation to characterize full-length HLA alleles can be overcome more easily by using third-generation sequencing methods in addition to traditional typing methods to produce more accurate HLA typing results. An advantage of our full-length HLA gene-sequencing protocol is that it generated 5′ and 3′ non-coding and intronic non-coding sequences that often are ignored, as well as providing the coding sequences that usually are used to characterize and classify HLA polymorphisms and investigate their associations with disease and transplantation. The polymorphisms and diversity of HLA non-coding sequences is additional beneficial information because non-coding sequences have regulatory roles in gene expression (55, 56) such as producing or interacting with regulatory microRNA (19, 54) and regulatory retrotransposons (57, 58). The HLA non-coding SNPs have been associated with disease and regulation of infection such as the SNP in the 3′UTR of HLA-C associated to plasma HIV copy number (19) and the SNP in the 3′UTR of HLA-DPB1 associated to acute GVHD (20). In this study, by comparing all the aligned DRB1^*^04 allele sequences, we identified new variations that may be related to RA susceptibility and resistance throughout the whole gene regions including a previously “undetermined region.” Within the group of DRB1^*^04 alleles certain shared epitopes at AA 70–74 have been associated with RA susceptibility (QRRAA, QKRAA) or resistance (QRRAE). In addition to these known associations, we identified two SNVs (SNV 9,139 and SNV 9,304) in intron 2 correlating with RA susceptibility. Although these non-coding SNVs are still under further investigation and their associations with RA need to be validated in comparative studies using RA patients and healthy subjects, it is evident that HLA-related disease analysis using the full-length HLA-gene sequences provides a new opportunity for the discovery of novel disease-related variations in the highly polymorphic HLA genes.

We undertook a comparative nucleotide analysis of the coding and the non-coding regions of all the HLA allele sequences and mapped their SNV and indel differences (Figure S2). We focused on the nucleotide diversity of the HLA-DPB1 sequences and found that there are no nucleotide differences among the alleles in the promoter-enhancer region (data not shown), but that 2.8 SNV/Kb on average were identified across the entire gene allelic sequences and that 4 SNV/Kb were identified in the non-coding regions between intron 2 and the 3′UTR region (Segment 2 of Figure 3A1). Phylogenetic analysis of all the HLA-DPB1 allelic sequences separated them into DP2 and DP5, two distinct groups that were previously described by phylogeny of peptide binding sequences and other evolutionary markers (46). Although the allele-specific polymorphisms observed in exon 2 appear to have been generated independently of the DP2 and DP5 subgroups, the SNV rs9277534 within the 3′UTR that was associated with the regulation of HLA-DPB1 gene expression (20) also was associated strongly with one or other of the two DP groups. The high expression rs9277534G grouped within DP5, whereas the low expression rs9277534A grouped within DP2. The role of this rs9277534 SNV in gene expression is important because it may provoke acute GVHD after unrelated hematopoietic cell transplantation (UR-HCT) in a different manner from the T-cell epitope (TCE) mismatching algorithm (59), reflecting exon 2 polymorphisms and structural differences of HLA-DP molecules between patient and donor and affect acute GVHD in HLA-DPB1 mismatched UR-HCT (60). Although phylogenetic trees of the 12,149 bp for whole gene region, the 2,668 bp from exon 3 to 3′UTR and the 264 bp for exon 2 were published previously (60), our present study has advanced the previous findings because we identified a genomic boundary between the DP2 and DP5 groups from the 19 HLA-DPB1 allele sequences.

In the present study, we used the computing program RepeatMasker to search for and identify novel retrotransposons in our full-length HLA-DRB1 sequences that could be useful polymorphic indel markers for investigating the evolution of ancestral haplotypes or be associated with diseases. We found DR-type specific indels of SINE, LINE, Medium reiterated repeat (MER) and Long terminal repeat (LTR) sequences that were mainly located within the introns 1, 2, and 5 of the HLA-DRB1 sequences. These findings on HLA retroelement indel polymorphisms complement and expand on the previous results of others (43, 44, 61).

## Conclusion

We determined, characterized and reclassified 253 full-length HLA allele sequences using both the third-generation SMRT and the second-generation Ion Torrent NGS technologies. These newly generated and classified reference sequences will help to genotype HLA genes to the full-length level and to accurately identify rare, novel and null alleles, which in turn should help to provide new information and knowledge for future population genetics, HLA-related disease, and transplantation studies. Finally, a much greater collection of high-quality, fully characterized full-length HLA allele sequences should be determined worldwide in many different populations.

## Data availability

The HLA allele sequences are available in GenBank/DDBJ/ENBL-EBI DNA databases and IPD-IMGT/HLA database. The detail information such as the accession numbers and official HLA allele names are indicated in the Table S7.

## Ethics statement

The study protocol was approved from the institutional review board of the Japan Marrow Donor Program (JMDP) and Tokai University (Application number: 15I-04) and informed consents were obtained from donors in accordance with the Declaration of Helsinki.

## Author contributions

SS, TS, and SR designed, set up some of the approaches, and carried out most of the experiments. JH analyzed the data. SI, AS, AM, JH, PB, and MK were involved in nucleotide sequencing. YO, AO, JS, SM, YM, and HI designed and supported the study. SS, TS, KO, and JK performed analysis and wrote the paper. All authors checked the final version of the paper.

### Conflict of interest statement

SR, JH and PB are employees of Pacific Biosciences, KO, MK and JS are employees of Tomy Digital Biology, and AM and HI are employees of GenoDive Pharma. The remaining authors declare that the research was conducted in the absence of any commercial or financial relationships that could be construed as a potential conflict of interest.

## Abbreviations

MHC: Major histocompatibility complex
HLA: Human leukocyte antigen
GVHD: Graft-versus-host disease
PCR: Polymerase chain reaction
RFLP: Restriction fragment polymorphisms
SSCP: Single strand conformation polymorphism
SSO: Sequence specific oligonucleotides
SSP: Sequence specific primers
SBT: Sequence based typing
UTR: Untranslated region
NGS: Next generation sequencing
SS-SBT: Super high resolution-single molecule-sequence-based typing
SNP: Single nucleotide polymorphism
SNV: Single nucleotide variation
SMRT: Single molecule, real-time
Indel: Insertion and deletion
RA: Rheumatoid arthritis
LAA: Long Amplicon Analysis
SD: Standard deviation
CDS: Coding sequence
SINE: Short interspersed nuclear element
LINE: Long interspersed nuclear element
HIV: Human Immunodeficiency Virus
UR-HCT: unrelated hematopoietic cell transplantation
TCE: T-cell epitope
MER: Medium reiterated repeat
LTR: Long terminal repeat
SNR: noise ratio
emPCR: Emulsion PCR
Blat: BLAST-like alignment tool
QV: quality value
SeaBass: Sequence Alignment Based Assigning Software.
