# Supplementary material for: Reference Grade Characterization of Polymorphisms in Full-Length HLA Class I and II Genes With Short-Read Sequencing on the ION PGM System and Long-Reads Generated by Single Molecule, Real-Time Sequencing on the PacBio Platform
Source: Front Immunol. 2018 Oct 4;9:2294. doi: 10.3389/fimmu.2018.02294 (PMC6180199; doi:10.3389/fimmu.2018.02294)
Supplement: Supplementary file 8 [file Presentation_1.PPTX]

## Slide 1
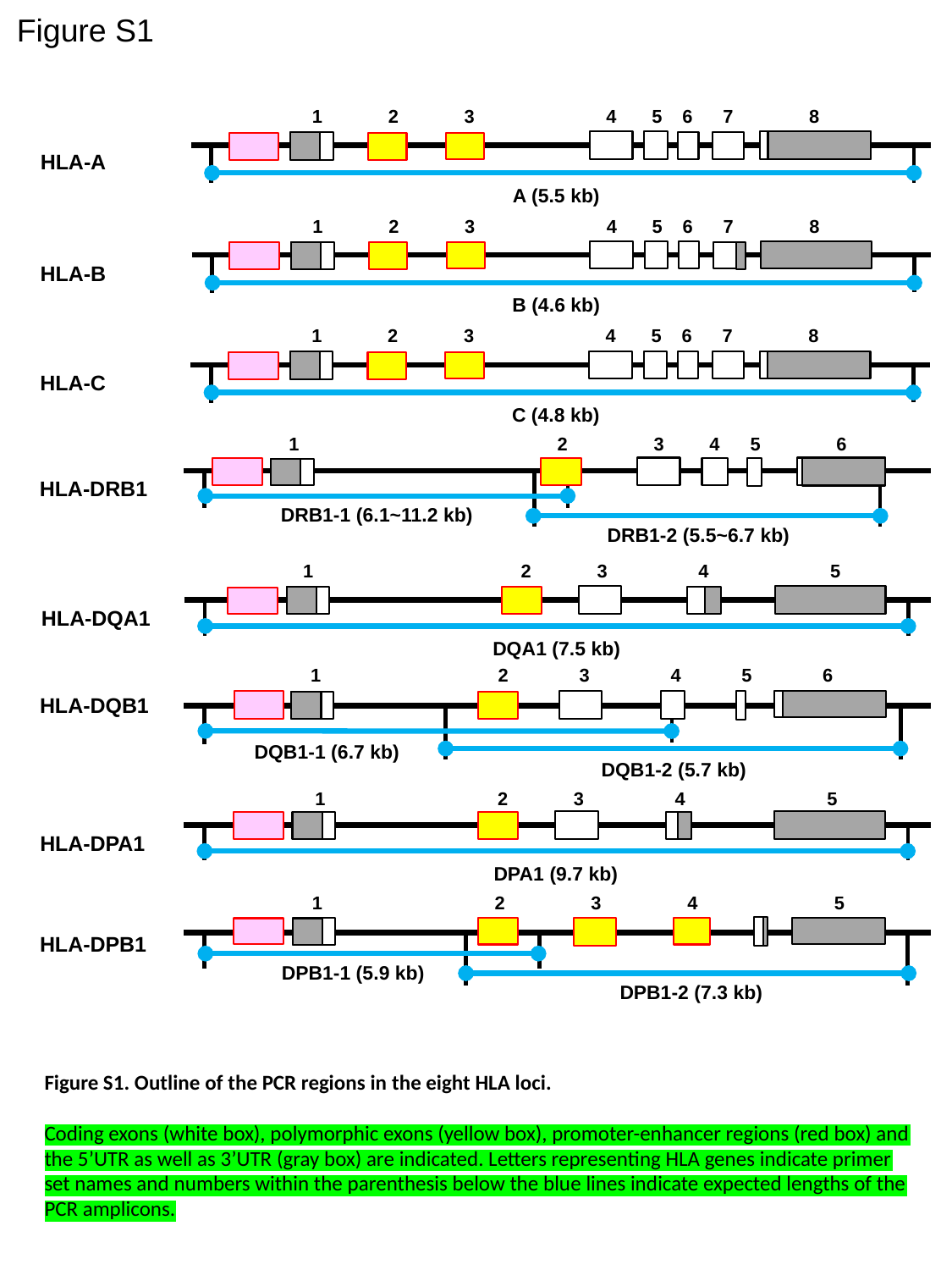

Figure S1
1 2 3 4 5 6 7 8
HLA-A
A (5.5 kb)
1 2 3 4 5 6 7 8
HLA-B
B (4.6 kb)
1 2 3 4 5 6 7 8
HLA-C
C (4.8 kb)
 1 2 3 4 5 6
HLA-DRB1
DRB1-1 (6.1~11.2 kb)
DRB1-2 (5.5~6.7 kb)
1 2 3 4 5
HLA-DQA1
DQA1 (7.5 kb)
1 2 3 4 5 6
HLA-DQB1
DQB1-1 (6.7 kb)
DQB1-2 (5.7 kb)
1 2 3 4 5
HLA-DPA1
DPA1 (9.7 kb)
 1 2 3 4 5
HLA-DPB1
DPB1-1 (5.9 kb)
DPB1-2 (7.3 kb)
Figure S1. Outline of the PCR regions in the eight HLA loci.
Coding exons (white box), polymorphic exons (yellow box), promoter-enhancer regions (red box) and the 5’UTR as well as 3’UTR (gray box) are indicated. Letters representing HLA genes indicate primer set names and numbers within the parenthesis below the blue lines indicate expected lengths of the PCR amplicons.
