# Supplementary material for: Reference Grade Characterization of Polymorphisms in Full-Length HLA Class I and II Genes With Short-Read Sequencing on the ION PGM System and Long-Reads Generated by Single Molecule, Real-Time Sequencing on the PacBio Platform
Source: Front Immunol. 2018 Oct 4;9:2294. doi: 10.3389/fimmu.2018.02294 (PMC6180199; doi:10.3389/fimmu.2018.02294)
Supplement: Supplementary file 9 [file Presentation_2.PPTX]

## Slide 1
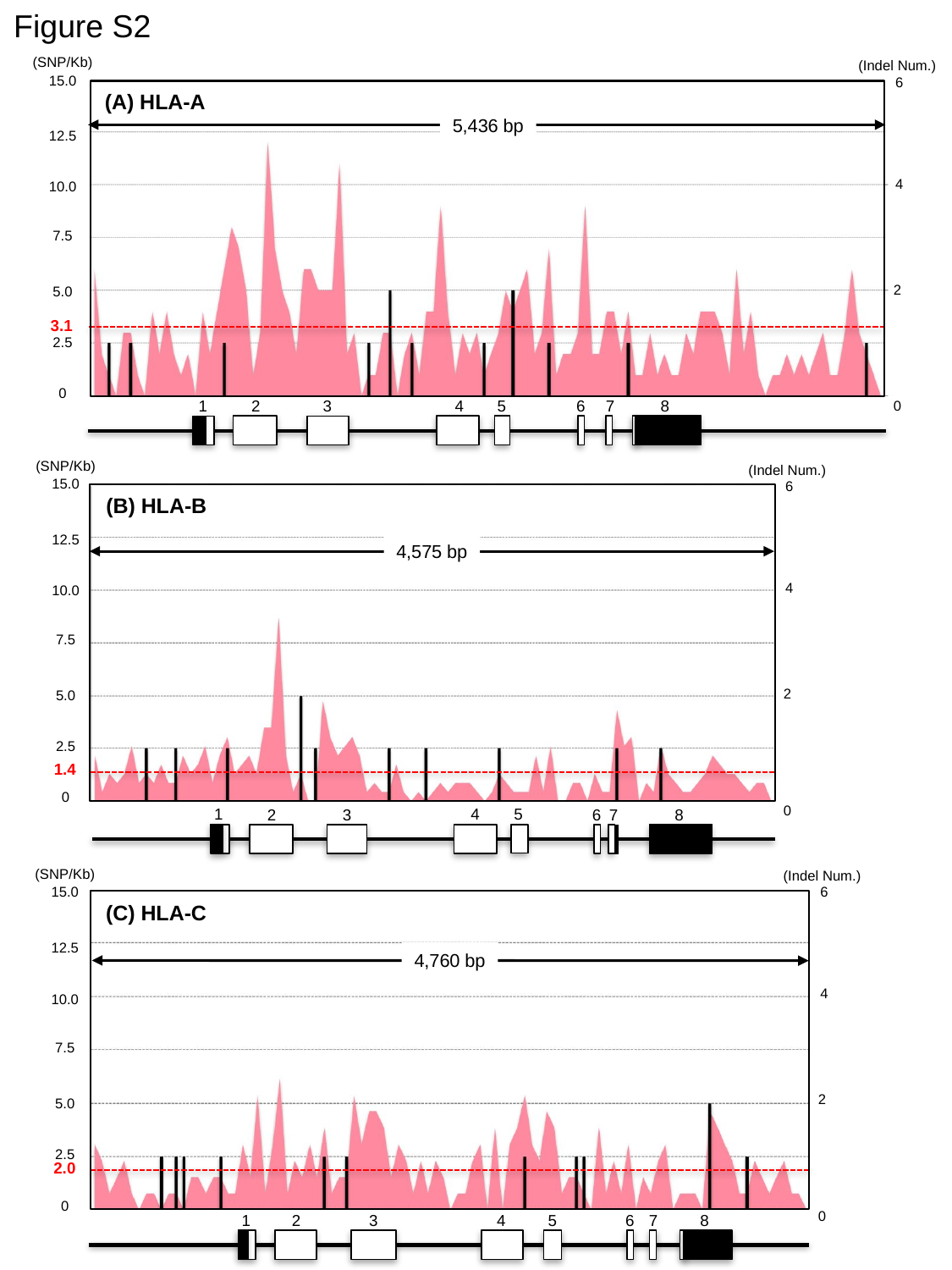

Figure S2
(SNP/Kb)
15.0
12.5
10.0
7.5
5.0
2.5
0
(Indel Num.)
 6
 4
2
0
(A) HLA-A
5,436 bp
3.1
1
4
5
6
7
3
8
2
(SNP/Kb)
15.0
12.5
10.0
7.5
5.0
2.5
0
(Indel Num.)
 6
 4
2
0
(B) HLA-B
4,575 bp
1.4
1
4
5
6
7
3
8
2
(SNP/Kb)
15.0
12.5
10.0
7.5
5.0
2.5
0
(Indel Num.)
 6
 4
2
0
(C) HLA-C
4,760 bp
2.0
1
4
5
6
7
3
8
2

## Slide 2
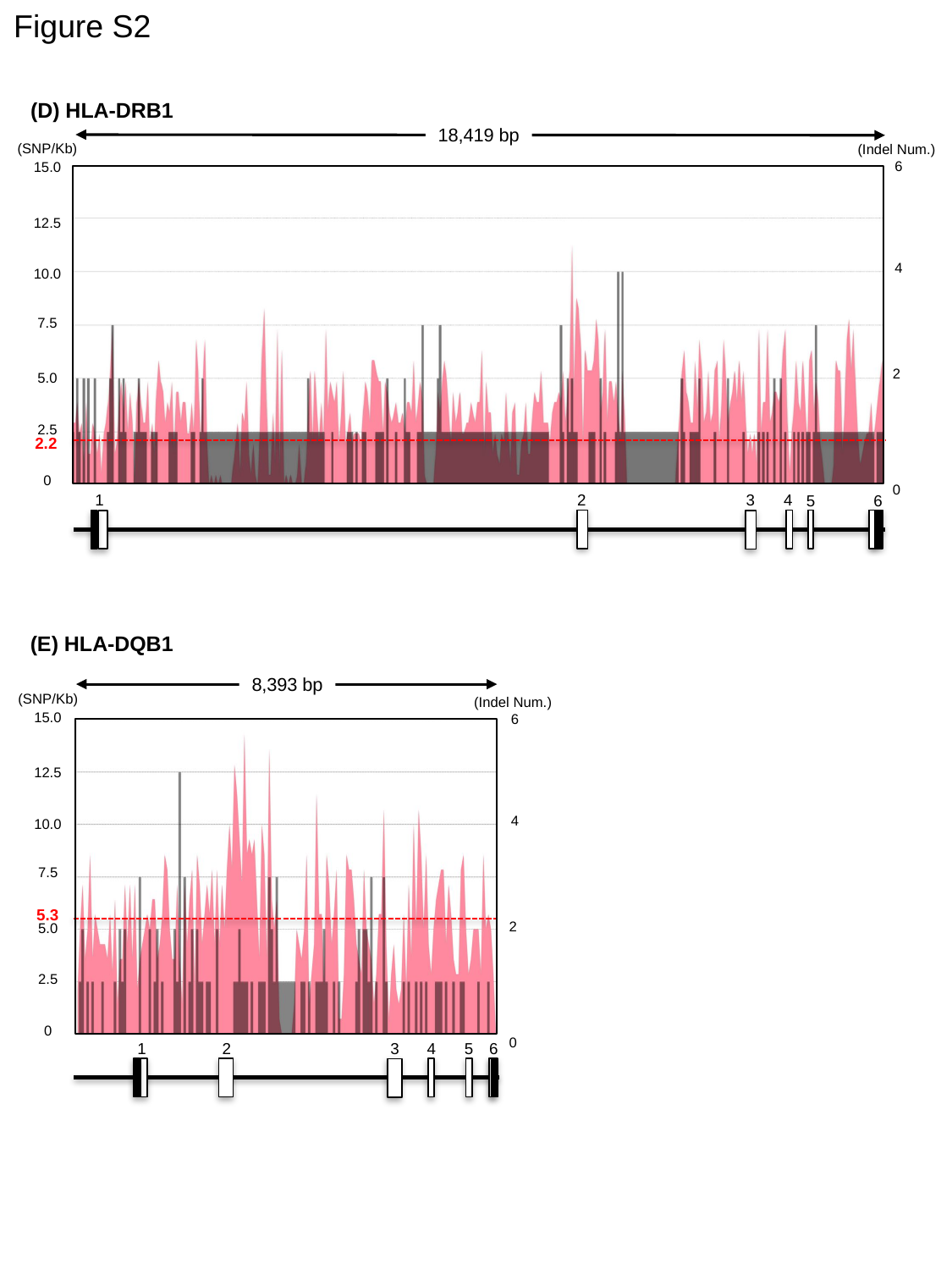

Figure S2
(D) HLA-DRB1
18,419 bp
(SNP/Kb)
15.0
12.5
10.0
7.5
5.0
2.5
0
(Indel Num.)
 6
 4
2
0
2.2
1
2
3
4
5
6
(E) HLA-DQB1
8,393 bp
(SNP/Kb)
15.0
12.5
10.0
7.5
5.0
2.5
0
(Indel Num.)
 6
 4
2
0
5.3
1
2
3
4
5
6

## Slide 3
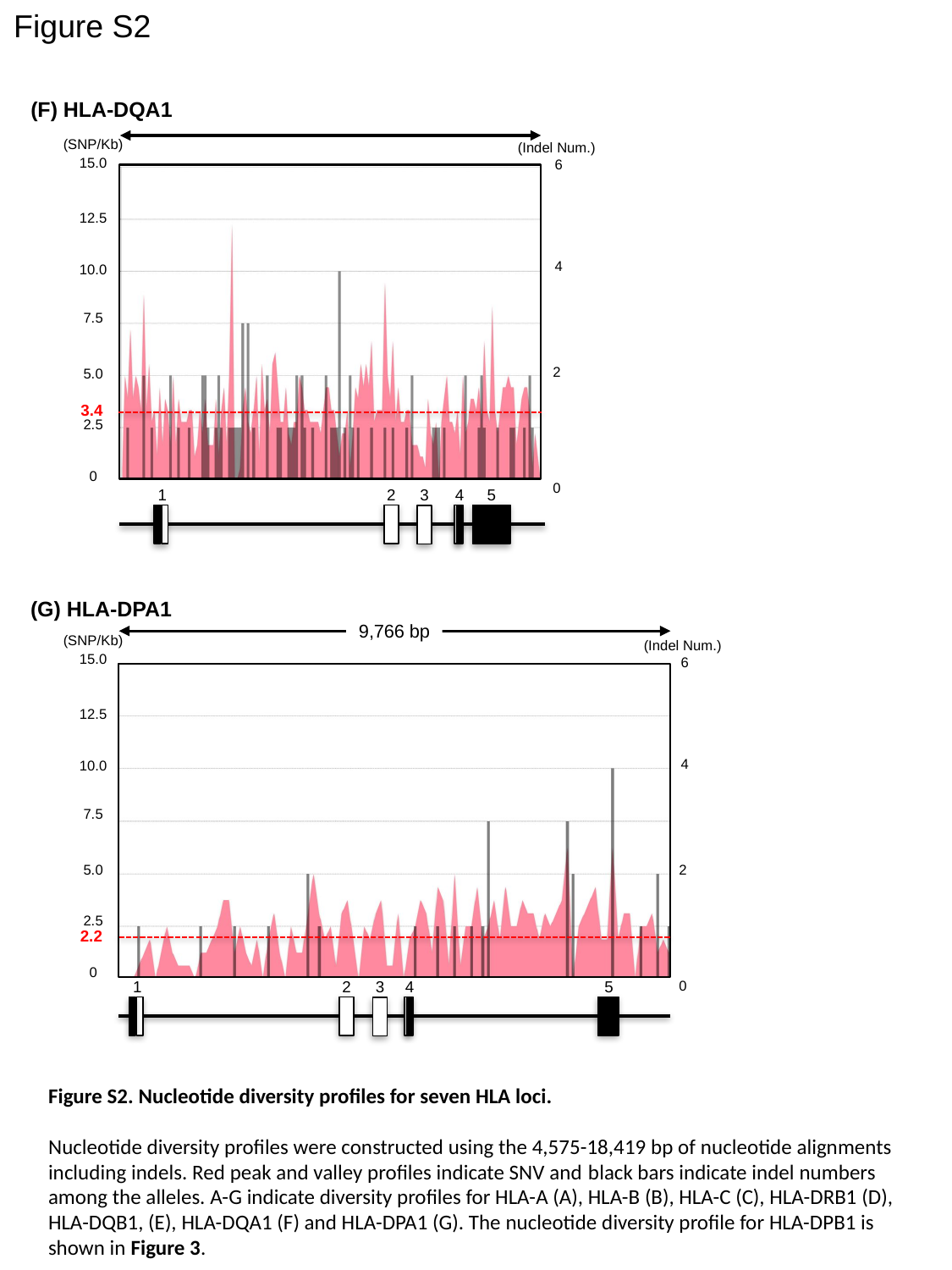

Figure S2
(F) HLA-DQA1
(SNP/Kb)
15.0
12.5
10.0
7.5
5.0
2.5
0
(Indel Num.)
 6
 4
2
0
3.4
1
2
3
4
5
(G) HLA-DPA1
9,766 bp
(SNP/Kb)
15.0
12.5
10.0
7.5
5.0
2.5
0
(Indel Num.)
 6
 4
2
0
2.2
1
2
3
4
5
Figure S2. Nucleotide diversity profiles for seven HLA loci.
Nucleotide diversity profiles were constructed using the 4,575-18,419 bp of nucleotide alignments including indels. Red peak and valley profiles indicate SNV and black bars indicate indel numbers among the alleles. A-G indicate diversity profiles for HLA-A (A), HLA-B (B), HLA-C (C), HLA-DRB1 (D), HLA-DQB1, (E), HLA-DQA1 (F) and HLA-DPA1 (G). The nucleotide diversity profile for HLA-DPB1 is shown in Figure 3.
