# Supplementary material for: Reference Grade Characterization of Polymorphisms in Full-Length HLA Class I and II Genes With Short-Read Sequencing on the ION PGM System and Long-Reads Generated by Single Molecule, Real-Time Sequencing on the PacBio Platform
Source: Front Immunol. 2018 Oct 4;9:2294. doi: 10.3389/fimmu.2018.02294 (PMC6180199; doi:10.3389/fimmu.2018.02294)
Supplement: Supplementary file 10 [file Presentation_3.PPTX]

## Slide 1
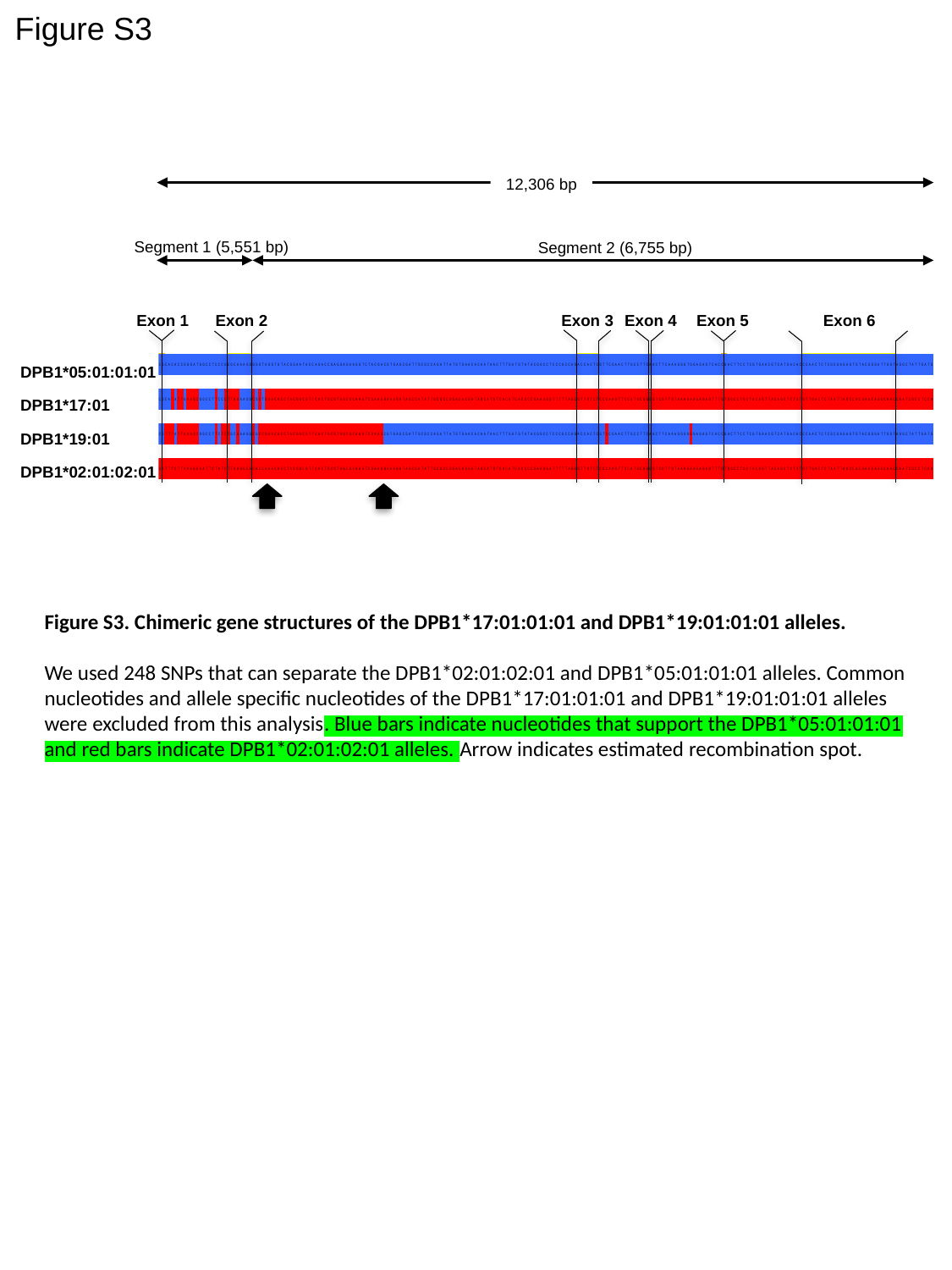

Figure S3
12,306 bp
Segment 1 (5,551 bp)
Segment 2 (6,755 bp)
Exon 6
Exon 2
Exon 3
Exon 4
Exon 1
Exon 5
DPB1*05:01:01:01
DPB1*17:01
DPB1*19:01
DPB1*02:01:02:01
Figure S3. Chimeric gene structures of the DPB1*17:01:01:01 and DPB1*19:01:01:01 alleles.
We used 248 SNPs that can separate the DPB1*02:01:02:01 and DPB1*05:01:01:01 alleles. Common nucleotides and allele specific nucleotides of the DPB1*17:01:01:01 and DPB1*19:01:01:01 alleles were excluded from this analysis. Blue bars indicate nucleotides that support the DPB1*05:01:01:01 and red bars indicate DPB1*02:01:02:01 alleles. Arrow indicates estimated recombination spot.
