# Supplementary material for: Reference Grade Characterization of Polymorphisms in Full-Length HLA Class I and II Genes With Short-Read Sequencing on the ION PGM System and Long-Reads Generated by Single Molecule, Real-Time Sequencing on the PacBio Platform
Source: Front Immunol. 2018 Oct 4;9:2294. doi: 10.3389/fimmu.2018.02294 (PMC6180199; doi:10.3389/fimmu.2018.02294)
Supplement: Supplementary file 11 [file Presentation_4.pdf]

**Figure S4**

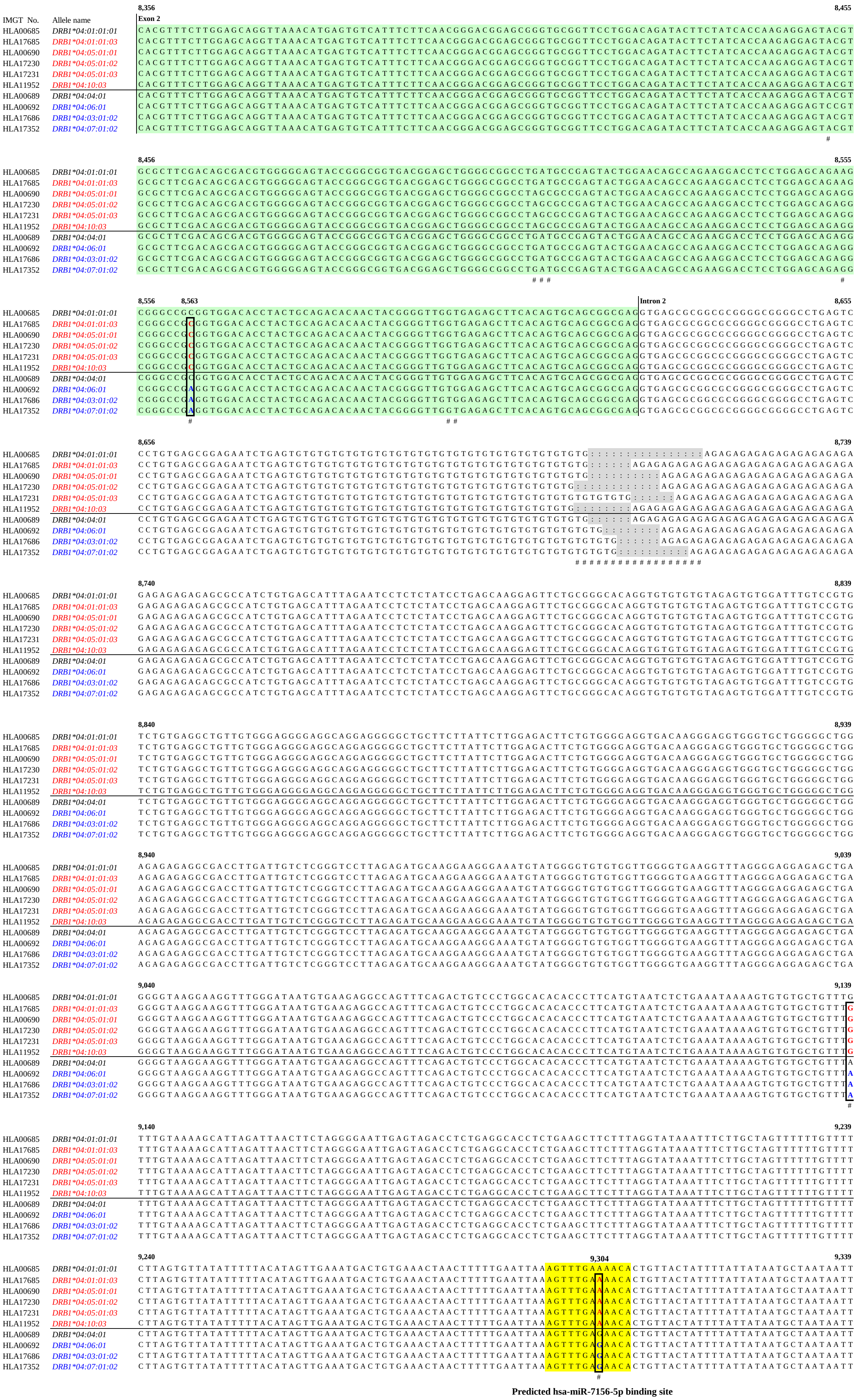


**Figure S4. Alignment of the DRB1*04 group of nucleotide sequences and location of a predicted hsa-miR-7156-5p binding site.**

The nucleotide positions are based on the previously published DRB1*04:01:01:01 (HLA00685) genomic sequence. This nucleotide alignment includes the entire exon 2 sequence and the first half of intron 2. RA-susceptible (red) and resistant (blue) alleles, exon 2 (green), indel (gray), predicted hsa-miR-7156-5p binding site (yellow) are shown in this figure. Black enclosures indicate three SNV (SNV 9,139 and SNV 9,304) sites. "#" indicates SNV site among the sequences. Nucleotide positions are bases on the sequence alignment tool of the IPD-IMGT/HLA database (https://www.ebi.ac.uk/ipd/imgt/hla/align.html) using the DRB1*04 group sequences described in this figure.
